# Arabic punctuation dataset

**DOI:** 10.1016/j.dib.2024.110118

**Published:** 2024-02-01

**Authors:** Sane Yagi, Ashraf Elnagar, Esra Yaghi

**Affiliations:** a Department of Foreign Languages, University of Sharjah, the United Arab Emirates; b Department of Computer Science, University of Sharjah, the United Arab Emirates; c Department of Linguistics, University of Waikato, Hamilton, New Zealand

**Keywords:** Punctuation corpus, Automatic punctuation, Sentence boundary identification, Topic and comment, Theme-rheme

## Abstract

Arabic, unlike many languages, suffers from punctuation inconsistency, posing a significant obstacle for Natural Language Processing (NLP). To address this, we present the Arabic Punctuation Dataset (APD), a large collection of annotated Modern Standard Arabic texts designed to train machine learning models in sentence boundary identification and punctuation prediction. APD leverages the “theme-rheme completion” principle, a grammatical feature closely linked to consistent punctuation placement. It consists of an annotated collection of Modern Standard Arabic (MSA) texts that encompass 312 million words in approximately 12 million sentences. It comprises three diverse components: Arabic Book Chapters (ABC): Manually annotated, non-fiction, book excerpts, constituting a gold-standard reference. Complete Book Translations (CBT): Parallel English–Arabic book translations with aligned sentence endings, ideal for machine translation training. Scrambled Sentences from the Arabic Component of the United Nations Parallel Corpus (SSAC-UNPC): Jumbled sentences for model training in automatic punctuation restoration. Beyond NLP, APD serves as a valuable resource for linguistics research, language learning, and real-time subtitling. Its authentic, grammar-based approach can enhance the readability and clarity of machine-generated text, opening doors for various applications such as automatic speech recognition, text summarization, and machine translation.

Specifications Table

**Table:** 

Subject	Computer Science
Specific subject area	Modern Standard Arabic (MSA) dataset annotated with sentence boundaries and theme-rheme-based punctuation.
Data format	Text files
Type of data	Text data
Data collection	The dataset components were collected as follows: (1) Arabic Book Chapters (ABC): One chapter was extracted randomly from each of 46 different books on the Hindawi Library [1]; each was published under the Creative Commons Attribution-NonCommercial 4.0 International License. All data from these books have been used in strict compliance with the terms of their licenses. (2) Complete Book Translations (CBT): From the same library, we obtained 60 full translations, each published under the same Creative Commons license. The translators manually punctuated these books based on the punctuation of the English source texts. (3) Scrambled Sentences from the Arabic Component of the United Nations Parallel Corpus (SSAC-UNPC). This part of the dataset consists of pre-processed, clean, and complete sentences that are not in their original order; they were randomly extracted from the UN Parallel Corpus which in turn had extracted them from the official records and parliamentary documents of the United Nations. The original documents were written and translated in the six official UN languages (Arabic, Chinese, English, French, Russian, and Spanish) between 1990 and 2014. The UN Corpus can be obtained from [2]. However, it is important to note that “The UN corpus is made available without warranty of any kind, explicit or implied. The United Nations specifically makes no warranties or representations as to the accuracy or completeness of the information contained in the UN corpus” [3]
Data source location	University of Sharjah, University City, Sharjah, United Arab Emirates. P.O.Box: 27272, Sharjah, UAE
Data accessibility	Repository name: Arabic Punctuation Dataset Data identification number: 10.17632/2pkxckwgs3.1 Direct URL to data: https://data.mendeley.com/datasets/2pkxckwgs3/1

## 1 Value of the Data

This dataset is valuable to:

• Developers of NLP applications in automatic speech recognition, text summarization, machine translation, and text generation; it is particularly useful for adding, restoring, and predicting punctuation marks in texts.
• Machine learning specialists; they can use it to train models and consequently improve the readability and clarity of machine-generated text.
• Linguists, teachers, and students of languages, as the APD consists of authentic texts that have been punctuated solely in accordance with the rules of grammar, rendering it with minimal idiosyncrasies and inconsistencies. The APD can be used to study both the structural patterns and the punctuation.
• Real-time subtitlers, as the APD may be used to instruct sentence boundary detection algorithms, convey tone and emotion, improve readability, disambiguate utterances, and synchronize with the audio track.

The dataset can be reused for:

• Training language models on the contextual nuances of when and where punctuation occurs within text to enable them to identify potential sentence boundaries.
• Extracting features such as type of punctuation mark, position, adjacent word types, and sentence structures associated with each type of mark to give models the ability to recognize and predict sentence boundaries.

## 2 Background

Punctuation serves numerous functions both in written and spoken language. It operates at various levels, including grammatical, semantic, pragmatic, prosodic, and rhetorical. Punctuation indicates the boundaries of clauses and sentences, as well as the relationships between words. It clarifies meaning, emphasizes words or phrases, and creates rhythm and pacing. Punctuation also indicates tone of voice, politeness, and formality. It creates emotional impact by emphasizing certain points over others. Additionally, punctuation is used to link written and spoken modes by indicating intonation, stress, pauses, and breaks in text-to-speech and speech-to-text.

Punctuation is also important in natural language processing. It clarifies meaning and resolves ambiguities, provides clues for understanding grammatical structures, ensures that machine translation output retain the intended meaning of the original text, operates as a feature in text classification tasks, and ensures that generated text sound natural.

What motivated the decision to develop this dataset is that automatic processing of Arabic faces challenges due in part to its erratic punctuation. We found it essential that there be a dataset specifically designed for punctuation to train artificial intelligence tools. In this dataset, sentence boundaries are determined by the rules of grammar rather than the writer's preference. This dataset will be used to train machine learning algorithms on automatically identifying and punctuating sentence boundaries in accordance with the rule of theme-rheme completion.

## 3 Data Description

This is a curated dataset, specifically designed to facilitate the study of punctuation. It has undergone rigorous manual annotation and verification based on sentence structure, with sentence boundaries clearly marked. To appreciate the effort invested in the manually annotated ABC component of the dataset, here is an example of one sentence-paragraph, punctuation-annotated, and translated into English.

The dataset is in three folders:

1. The ABC component of the Arabic Punctuation Dataset: This folder features the manually annotated punctuation gold standard; it has 46 text files, amounting to 2.08 MB. It consists of one chapter extracted from each of 46 non-fiction books by 38 authors from 21 different fields of study. It consists of 149K words in 13K sentences.
2. The CBT component: This folder has 1085 text files in 60 subfolders, totaling 33.7MB. Each subfolder contains the full text of a complete book translation, inside which are text files that represent chapters or sections in the book. The book had been rendered from English into Arabic independently of this project. The punctuation of translated books, we found, mirrors the English source language text; i.e., the sentence terminals in these Arabic texts follow the rules of English. In this folder are close to 3M words in more than 170K properly punctuated sentences.
3. The SSAC-UNPC component: This folder is a 3.2 GB collection of textual content that constitutes the third part of the Arabic Punctuation Dataset. It has 11.7M disconnected, disordered, complete sentences in one text file. These scrambled sentences are complete and clean; they were extracted from the predominantly legal Arabic subcorpus of the United Nations Parallel Corpus (UNPC). The punctuation here is authentic. It was done by the UN translators as part of their work. We consider this to be an excellent punctuation corpus because it mirrors the rule-governed punctuation of the English source documents, especially in relation to sentence terminals. These scrambled sentences total more than 309M words.

The three folders make up the Arabic Punctuation Dataset. As indicated in the table below, the APD is more than 312M words, in close to 12M sentences.

Notice that sentence length varies as it reflects the nature of the texts in the dataset components, with the non-fiction prose in ABC being the shortest and the legal text sentences in SSAC-UNPC being the longest. Paragraphs, on the other hand, are significantly longer in the authentic Arabic texts of the ABC than in the English to Arabic translations in the CBT.

In terms of punctuation, the APD consists of texts with the following punctuation characteristics:

Notice that in the CBT and SSAC-UNPC datasets, commas and full stops have the highest and second-highest frequencies, respectively, but their order is reversed in the ABC dataset. This variation is partly a result of the conscious decision of the annotators to focus on sentence termination, often leading to the replacement of commas with full stops.

In terms of relative frequency of sentence terminal marks, the full stop is by far the most frequent, the question mark is in second position, and the least frequent is the exclamation mark. This is most likely the predominant pattern in other languages too since the expression of emotions is less prevalent in texts than the communication of ideas. It is particularly true in the APD because it is composed of non-fiction texts.

## 4 Experimental Design, Materials and Methods

Arabic texts may exhibit a scarcity of punctuation, misuse it, or employ it inconsistently, despite its crucial role in conveying meaning, structure, and tone. To address this problem and to facilitate the training of machine learning models on correct punctuation, it is necessary to construct a dataset that is dedicated to punctuation alone and whose punctuation is non-controversial.

At the core of Arabic punctuation irregularities is the decision of where to place a sentence terminal. School teaches that a sentence is “a combination of words which is complete as expressing a thought, and in writing is marked at the close by a period, or full point” [4, p.14]. School does not teach how to recognize when a ‘complete’ thought has ended [5]. It does not explain what constitutes a complete thought, how to identify one, or how to distinguish it from an incomplete thought. In other words, the definition is vague and subjective.

While Bloomfield's influence is evident in modern linguistics, with many adhering to his view that “each sentence is an independent linguistic form, not included by virtue of any grammatical construction in any larger linguistic form” [6, p.170], it's crucial to also consider the perspective of text linguistics. This approach emphasizes the interconnectedness of sentences within a larger textual and communicative context, which is particularly relevant when considering the role of punctuation in demarcating sentence boundaries. In English and Arabic, diverse grammatical structures serve to characterize sentences and differentiate between sentence types. However, these sentences do not exist in isolation. They are part of a larger discourse, and their meaning and interpretation can often depend on their context within this discourse. In both languages, sentences are not only demarcated through modulation, where pitch-based secondary phonemes are utilized to indicate sentence boundaries, but also through their coherence and relation to the surrounding text. Sentence terminal punctuation is, therefore, critical.

Sentences are functionally categorized into declarative, interrogative, imperative, and exclamative forms, and they are structurally classified into simple, compound, and complex types. A sentence typically comprises a singular subject-predicate unit unless it falls under the compound category.

### 4.1 Concept of sentence in the Arabic linguistic tradition

Arab linguists have recognized the concept of sentence since the time of Sībawayhi (d. 796 C.E.). However, some of these linguists have used the terms kalām and jumla to refer to this concept. Ibn Hishām (d. 1360 C.E.) defined kalām in [7] as a saying that is useful or communicative and that denotes a meaning after which it is proper to be silent. Jumla, on the other hand, is a predicative construction that may not necessarily be intelligibly uttered on its own. Ibn Hishām specifically pointed out that every kalām is a jumla, but not every jumla is a kalām.

Ibn Hishām also introduced the concept of an enlarged predicate construction, in which the predicate itself is a jumla and its predicate is another jumla. Consequently, these constructions, he classified into two types: jumla kubrā, which is a complex sentence with a predicate that is itself a jumla, and jumla ṣughrā, which is a predicate clause. This division is based on the relationship between the different components within these constructions.

When Arabs adopted their punctuation system from the West, they abandoned the traditional concepts of kalām and jumla and took up the Eurocentric definition of sentence as the expression of a complete thought. This has led to some confusion among litterateurs as to what constitutes a complete thought, whether it is the ‘sentence’ or the ‘paragraph’; this resulted in the two terms becoming practically interchangeable. In our study that investigated whether Arabic punctuation was rule-governed, sentence and paragraph lengths in several genres were contrasted and the conclusion was that “even well-versed writers of Arabic cannot agree on where to place a sentence terminal”; therefore, the study concluded that Arabic punctuation is in a state of flux [8].

### 4.2 Grammar-based sentence boundary identification

We advocate that punctuation rules be based on grammar rather than meaning. We propose two central punctuation rules: (1) Sentences should be terminated upon the completion of the ‘topic and comment’ in the parlance of the Prague School, ‘theme and rheme’ in the language of Systemic Functional Linguistics, and ‘musnad and musnad ilayh’ in the terminology of Classical Arabic Grammar. (2) Conjunctions of the type of *wa* are to be disregarded in the determination of sentence boundary. The grammar knowledge required for these two rules, and for sentence boundary identification at large, is integral to the language competence of all native speakers; it is part of their subconscious intuitive knowledge of their own language. Everybody knows when an attribution has been made. Sentence termination requires no knowledge of the metalanguage of linguists.

The theme, topic, or musnad ilayh is the sentence element that refers to an entity about which the speaker or writer is talking, while the rheme, comment, or musnad are the element that represents the new information that the speaker/writer wishes to attribute to the identified entity. Hence forth, we will refer to the first element as theme, and the second element as rheme.

The sentence may be viewed as an attribution of a state or event to a referring expression. It quite often attributes a physical, mental, behavioral, verbal, relational, or existential process to the referent that the theme identifies. The rheme that represents the state or event may take place at some circumstance and may involve one or more participants. In terms of involvement, the participants may be directly involved in the state or event in the capacity of actor or goal of the process, sensor of a phenomenon, sayer of something, token of a certain value, carrier of an attribute, object of identification, or topic of existence. A participant may also be indirectly involved in the event by being the beneficiary or the element that specifies the range of the process.

A sentence may take the most basic form of a theme-rheme configuration to communicate a simple thought such as, “Sarah is good”, or an expanded thought, where one or both sentence elements are elaborated, extended, or enhanced, as in this complex sentence: “The blonde Sarah you met downtown is a skilled architect and civil engineer who can design and build the house of your dreams”. Clause expansion is usually performed paratactically by linking constituents of equal status (e.g., a skilled architect *and* civil engineer) or hypotactically by binding elements of unequal status together where one is free whilst the other is dependent on it (e.g., civil engineer *who* can design and build the house of your dreams).

To avoid the use of folk linguistic terms [9], let ‘sentence’ be a group of words that consists minimally of two constituents, a theme and a rheme, but may optionally include other words that expand the theme and/or rheme or that may link with them paratactically (by coordination) or hypotactically (by subordination). Let the sentence be, in other words, synonymous with an independent clause or an expanded clause.

When a clause enters into construction with a similar unit, the two form an expanded structure, a clause complex, where there is an extended noun group, or an extended verb group, or both. Expansion takes three forms: (a) elaboration by exposition, exemplification, or clarification; (b) extension by addition, replacement, or by offering an alternative; and (c) enhancement by time, place, manner, cause, or condition qualification. An expanded clause is to be terminated by a sentence terminal: a full stop, question mark, or exclamation mark, whichever is contextually appropriate.

Expansion may also be done paratactically or hypotactically. According to [9, p.452] parataxis is “the linking of elements of equal status... Both the initiating and the continuing element are free, in the sense that each could stand as a functioning whole… [Hypotaxis is] the binding of elements of unequal status. The dominant element is free, but the dependent element is not”.

In terms of punctuation, the paratactic elements constitute independent clauses and each of them would be terminated by a sentence terminal. The hypotactic elements, on the other hand, constitute a single clause with one being free and independent while the others dependent on it; therefore, the hypotactic elements together with the independent clause would constitute a clause complex and would, therefore, be terminated by a single sentence terminal.

The dataset discussed here conforms to this understanding of ‘sentence’ and it particularly adheres to the two rules that define the sentence structure as a theme and rheme and that ignores the conjunction *wa* in sentence boundary identification.

### 4.3 Annotation methodology

With this understanding of ‘sentence’, two annotators of the ABC component of our APD dataset were instructed to read a book chapter and reflect on where each sentence should be terminated in the light of the Annotation Guidelines [11].

The concept of ‘sentence’ was operationalized as follows:

• One word or group of words that is informative independently of what precedes or follows.
• Ignoring the effects of reference and conjunctions, the sentence is autonomous and self-sufficient.
• It can be uttered in isolation without losing its power of predication, assertion, existentiality, imperativeness, interrogativeness, or exclamation.
• Subordination is only modification.
• Commas and other intra-sentential punctuation are to be left unchanged since they are primarily discretionary.

In order to determine the sentence boundaries within the ABC component of our APD, we directed two annotators to read each book chapter and mark the end of every sentence with an appropriate punctuation mark guided by their own verbalization [10] and adhering to the Annotation Guidelines (Table 1).

**Table 1:** Example of punctuation-annotation.

English translated	Punctuation-annotated	Original text
Education in ancient Greece was aimed at creating a strong body prepared for wars, for the defense of the country and for conquest; their schools were a factory for this purpose. As a result, the goal of education in Athens became the creation of an intelligentsia concerned with philosophy and the understanding of nature and the supernatural, so schools were established. In them, Plato and Aristotle taught, adopting this pattern to achieve this purpose. Then came the era of the Romans; their most important goals were military education, with its arts, systems and strategies; and the teaching of rhetoric with emphasis on eloquence in orations. Their schools were designed for these two purposes. In the Middle Ages, the overwhelming trend was that of religiosity; the school took on this character, and everything was taught for the service of religion, even linguistics and the cognitive sciences.	لقد كانت التربية في عهد اليونان الأقدمين ترمي إلى خلق جسم قوي معد للحروب وللدفاع عن البلاد وللفتوح**؛** فكانت مدارسهم مصنعًا لتأدية هذا الغرض**.** وتحول غرض التربية في أثينا إلى إيجاد طبقة عقلية تُعنَى بالفلسفة وفهم الطبيعة وما وراء الطبيعة**،** فأنشئت المدارس**.** يعلم فيها أفلاطون وأرسطو على هذا النمط لتحقيق هذا الغرض**.** وجاء عهد الرومان**؛** فكان أهم غرض رئيسي لهم التعليم الحربي في فنونه ونظمه وترتيباته**،** والتعليم البلاغي في تحرير الخطب وفصاحة اللسان**.** فكانت مدارسهم تُعد لهذين الغرضين**.** وفي العصور الوسطى غمرت الناس الموجة الدينية**؛** فصبغت المدرسة هذه الصبغة**،** وكان كل شيء يُعلَّم لغرض الدين**،** حتى العلوم اللسانية والعلوم العقلية	لقد كانت التربية في عهد اليونان الأقدمين ترمي إلى خلق جسم قوي معد للحروب وللدفاع عن البلاد وللفتوح**،** فكانت مدارسهم مصنعًا لتأدية هذا الغرض**،** وتحول غرض التربية في أثينا إلى إيجاد طبقة عقلية تُعنَى بالفلسفة وفهم الطبيعة وما وراء الطبيعة**،** فأنشئت المدارس يعلم فيها أفلاطون وأرسطو على هذا النمط لتحقيق هذا الغرض**،** وجاء عهد الرومان فكان أهم غرض رئيسي لهم التعليم الحربي في فنونه ونظمه وترتيباته**،** والتعليم البلاغي في تحرير الخطب وفصاحة اللسان**،** فكانت مدارسهم تُعد لهذين الغرضين**،** وفي العصور الوسطى غمرت الناس الموجة الدينية فصبغت المدرسة هذه الصبغة**،** وكان كل شيء يُعلَّم لغرض الدين**،** حتى العلوم اللسانية والعلوم العقلية**.**

The annotators were trained on sentence boundary identification with the aid of the Guidelines and an expert coach. Then, they independently punctuated one book chapter. Their punctuation was reviewed by the coach, and points of disagreement were identified and discussed with the respective annotator. This procedure was repeated four more times, each time on a different book chapter. Once the inter-annotator agreement exceeded 0.80, the training stopped, and the annotation task began in full. At the end of the annotation task, Cohen's Kappa was calculated for the whole ABC dataset to assess agreement between the two annotators (κ = 0.89). Points of disagreement were given to the coach to make the final punctuation decision based on the Punctuation Guidelines (Table 2).

**Table 2:** APD statistics.

Attribute	ABC	CBT	SSAC-UNPC	Total
Titles	46	60		
Words	148,982	2,917,152	309,630,104	312,696,238
Sentences	13,142	170,256	11,700,400	11,883,798
Words/Sentence	17.04	22.85	26.46	22
Paragraphs	1,167	75,098		76,265
Words/Paragraph	127.66	38.84		83
Sentences/Paragraph	11.26	2.27		7

The CBT and SSAC-UNPC parts of the APD were not originally intended as punctuation corpora, but we used them for training machine learning algorithms because their punctuation is authentic and rule-governed. It is our view that punctuation is a natural byproduct of the translation process; it closely mirrors the source text's punctuation. Translators do not deviate from sentence boundary mirroring unless to emphasize specific nuances or to solve particular structural problems (Table 3).

**Table 3:** Percentage of punctuation marks in APD.

Punctuation	ABC	CBT	SSAC-UNPC	Total
Question marks	16.91	4.24	0.19	0.27
Colons	15.48	7.47	1.99	2.09
Commas	27.22	47.65	52.54	52.44
Semicolons	0.63	4.69	5.55	5.53
Exclamation marks	1.10	1.77	0.00250	0.03
Full stops	38.66	34.20	39.72	39.63

Acknowledging the lack of universal benchmarks for evaluating Arabic punctuation accuracy, Yagi et al. [8] conducted a cross-linguistic study to compare Arabic punctuation patterns with English rule-based punctuation. The results showed a strong positive correlation between the sentence sizes in the English source texts and their Arabic translations. Hence, sentence size in these two components of the APD is not drastically different from sentence size in the ABC component. Sentences in all three parts of the APD range between 17 and 26 words in length, compared to sentences in Arabic editorials, books, and linguistics and literature abstracts where they range from 41 to 56 words/sentence [8].

The CBT and SSAC-UNPC are similar to the ABC in that they follow the theme-rheme based punctuation criterion; this makes their punctuation rule-based.

The APD is a valuable resource for any corpus linguists and systemic linguists who might be interested in studying MSA text punctuation. It can be used to develop and evaluate computational models of punctuation prediction and to investigate the relationship between punctuation and other linguistic phenomena, such as syntax, semantics, and pragmatics.

## Limitations

This dataset prioritizes sentence boundary identification over fine-grained punctuation differentiation. It was a conscious decision that allowed annotators to focus on one primary task, allowing them to dedicate their full attention to the task; this decision may be perceived as a limitation. However, it is motivated by the recognition that accurate sentence segmentation remains a primary challenge in Arabic natural language processing. While other punctuation marks are included in the annotation of our dataset, their determination was less rigorous. Differentiation between semicolons and commas, for example, was left to annotator discretion. For researchers seeking to analyze nuanced punctuation usage, this dataset might necessitate further annotation. For those solely interested in sentence parsing, this dataset offers a valuable resource. However, for researchers interested in specific punctuation marks, they will only need to focus on semicolons and exclamation marks, which are of low frequency anyway.

## Ethics Statement

Ethical and legal considerations were of paramount importance in the compilation of this dataset. All data used in this study was sourced and processed in full compliance with copyright laws, licenses, and ethical standards.

## CRediT authorship contribution statement

**Sane Yagi:** Conceptualization, Methodology, Resources, Visualization, Writing – original draft. **Ashraf Elnagar:** Methodology, Data curation, Software, Writing – review & editing. **Esra Yaghi:** Resources, Data curation, Writing – review & editing.

## Data Availability

Arabic Punctuation Dataset (Original data) (Mendeley Data)
